# Anti-VEGF Therapy and the Retina: An Update

**DOI:** 10.1155/2015/627674

**Published:** 2015-08-31

**Authors:** Vikas Tah, Harry O. Orlans, Jonathan Hyer, Edward Casswell, Nizar Din, Vishnu Sri Shanmuganathan, Louise Ramskold, Saruban Pasu

**Affiliations:** ^1^The Royal Berkshire NHS Foundation Trust, Craven Road, Reading RG1 5AN, UK; ^2^Moorfields Eye Hospital NHS Foundation Trust, 162 City Road, London EC1V 2PD, UK; ^3^St George's University of London, Cranmer Terrace, London SW17 0RE, UK

## Abstract

Ocular angiogenesis and macular oedema are major causes of sight loss across the world. Aberrant neovascularisation, which may arise secondary to numerous disease processes, can result in reduced vision as a result of oedema, haemorrhage, and scarring. The development of antivascular endothelial growth factor (anti-VEGF) agents has revolutionised the treatment of retinal vasogenic conditions. These drugs are now commonly employed for the treatment of a plethora of ocular pathologies including choroidal neovascularisation, diabetic macular oedema, and retinal vein occlusion to name a few. In this paper, we will explore the current use of anti-VEGF in a variety of retinal diseases and the impact that these medications have had on visual outcome for patients.

## 1. Introduction

Ocular angiogenesis is a cause of severe worldwide visual loss and ocular morbidity. However, the development of antivascular endothelial growth factor (anti-VEGF) has revolutionised the treatment of a plethora of ocular angiogenic disease processes. It has become the favoured therapy for conditions such as choroidal neovascularisation, diabetic macula oedema, vein occlusions, myopic choroidal neovascularisation, and retinopathy of prematurity to name a few [1]. In 2013, Avastin (bevacizumab) and Lucentis (ranibizumab) were ranked 9th and 19th, respectively, in terms of top global sales of pharmaceutical products emphasising their impact in medicine as a whole [2].

It could be argued that the evolution of anti-VEGF therapy can be traced back to 1948, where Michaelson hypothesised that a diffusible, hypoxia-induced, angiogenic “factor X” was responsible for iris and retinal neovascularisation associated with ischaemic retinopathies. Decades later, a candidate glycoprotein was partially described and termed vascular permeability factor. Further research extended our understanding of this endothelial cell-specific glycoprotein; however, it was only in 1989; Leung et al. isolated an endothelial mitogen from pituitary follicular cells leading to it being branded, vascular endothelial growth factor (VEGF). At the same time, Keck et al. discovered a tumour-derived factor named vascular permeability factor (VPF), which was responsible for inducing vascular permeability. Since then, researchers strongly suggest that this diffusible, hypoxia-induced, endothelial cell-specific factor VEGF conceivably represents Michelson's retinal tissue “factor X” [3]. Subsequent sequencing of these two genes led to the realisation that the factors were in fact identical. In a further study, where the retinas of primates were rendered ischaemic by laser photocoagulation of the veins, neovascularisation of the iris developed suggesting the presence of a diffusible molecule. That diffusible molecule was found to be VEGF mRNA [4]. Furthermore, elevated levels of VEGF in ocular fluids from patients with active neovascular ocular disease were found compared with ocular fluids with no vascularisation. All the evidence of angiogenesis points to the role of VEGF in ocular neovascularisation [5].

In 1997, Genetech initiated phase 1 trials for the development of an anti-VEGF molecule named Avastin (bevacizumab). Subsequent successful results from phases 2 and 3 trials led to FDA approval in February 2004 for the treatment of colon cancer in combination with chemotherapy [6]. Furthermore, with the knowledge that VEGF played a significant role in neovascular AMD, FDA approved pegaptanib (Macugen) was created, making it the first antiangiogenic therapy for ocular neovascularisation [7]. After approval of bevacizumab for cancer therapy and VEGF's role in wet AMD, systemic IV bevacizumab was used as an off-label medication [8]. Ophthalmologists soon began to inject bevacizumab into the vitreous cavity leading to positive results virtually eliminating the systemic side effects [9].

Believing that bevacizumab would not efficiently diffuse through the retina to reach the choroid, Genetech decided to generate a truncated alternative molecule. Ranibizumab (Lucentis) was determined effective by two pivotal trials: the MARINA (minimally classic/occult trial of the anti-VEGF antibody ranibizumab in the treatment of neovascular AMD) and ANCHOR (anti-VEGF antibody for the treatment of predominantly classic choroidal neovascularisation in AMD) trials. Both of these trials were the first phase 3 trials to show improvement in visual outcomes for all forms of choroidal neovascularisation and were given FDA approval in 2006 [10, 11].

A recent anti-VEGF strategy, developed by Regeneron, consisted of a chimeric fusion protein that acted as a decoy receptor to sequester VEGF and thereby block its biological effects. Aflibercept was developed to improve the pharmacokinetics of VEGF binding with reduced frequency of dosing. Based on the VIEW study, aflibercept was approved by the FDA in November 2011 [12].

In this paper, we will explore the current indications of anti-VEGF in a variety of ocular angiogenic conditions that has changed the visual outcomes of patients.

## 2. DMO

Diabetic macular oedema (DMO or DME) is the leading cause of visual impairment in patients aged 20 to 74 and represents a significant burden of disease with the increasing incidence and prevalence of diabetes worldwide [13, 14]. The development of DMO occurs as a result of vascular endothelial damage with breakdown of the blood-retinal barrier. Hypoxia caused by microvascular disease stimulates the release of vascular endothelial growth factor-A (VEGF-A) which is a major contributor to this vascular permeability and angiogenesis [15].

Ranibizumab (IVR) (Lucentis; Genentech Inc.; marketed by Novartis in Europe) belongs to a class of drugs that block the action of VEGF-A, thus reducing oedema and stabilising or improving vision. It has a European marketing authorisation for the “treatment of visual impairment due to macular oedema in adults” and has been recommended by the National Institute for Health and Care Excellence (NICE) in April 2013 as a treatment option if the eye has a central retinal thickness of 400 micrometres (*μ*m) or more at the start of treatment [16]. This is also conditional on the manufacturer providing IVR with the discount agreed upon in the patient access scheme. The four randomised controlled trials (RCTs) submitted to NICE as evidence for clinical effectiveness included RESTORE (ranibizumab monotherapy or combined with laser versus laser monotherapy for diabetic macular edema), Diabetic Retinopathy Clinical Research Network Protocol I (DRCR.net), RESOLVE (Safety and Efficacy of Ranibizumab in Diabetic Macular Edema), and READ-2 (Ranibizumab for Edema of the mAcula in Diabetes) [17–21].

The RESTORE and DRCR.net (Diabetic Retinopathy Clinical Research Network) RCTs received detailed attention whilst the others were judged to be of less direct relevance. The RESTORE trial concluded IVR monotherapy and combined with laser provided superior visual acuity (measured with Early Treatment Diabetic Retinopathy Study- (ETDRS-) like charts) gain over patients treated with laser alone. At month 12, the visual acuity of eyes randomised to IVR monotherapy rose by a mean average of 6.1 letters and eyes randomised to IVR plus laser photocoagulation gained a mean average of 5.9 letters. Eyes randomised to laser photocoagulation alone gained fewer letters (0.8) than eyes randomised to either IVR-containing arm (*P* < 0.001). Mean central thickness was significantly reduced from baseline with IVR (−118.7 *μ*m) and IVR plus laser (−128.3 *μ*m) versus laser (−61.3 *μ*m); both *P* < 0.001. IVR monotherapy combined with laser had a safety profile in DMO similar to that in age-related macular degeneration with no endophthalmitis cases reported and one reported patient with increased intraocular pressure in each IVR arm.

The DRCR.net trial was funded by the US National Institute of Health and was a multicentre randomised (by eye rather than participant) to sham injection and prompt laser (*n* = 293), IVR and prompt laser (*n* = 187), IVR and deferred (≥24 weeks) laser (*n* = 188), or triamcinolone and prompt laser (*n* = 186). Only the results of 12-month follow-up were available for the NICE technology appraisal, although two-year follow-up is now published [18]. Compared with the sham injection plus prompt laser group, the mean change in the visual acuity (ETDRS) letter score from baseline was 3.7 letters greater in the IVR plus prompt laser group, 5.8 letters greater in the IVR plus deferred laser group, and 1.5 letters worse in the triamcinolone plus prompt laser group. Three eyes had injection-related endophthalmitis in the IVR groups. Although none of the RCTs of IVR in DMO were designed primarily to assess safety outcomes, no significant differences were observed between arms in the frequency of ocular and nonocular adverse events.

The approval of IVR by the U.S. Food and Drug Administration (FDA) for DMO was based on Genentech's phase III trials, RIDE, and RISE [22]. These trials were identically designed, parallel, double-blind, multicentre, three-year trials. Subjects received 0.3 mg IVR (*n* = 250), 0.5 mg IVR (*n* = 252), or sham injection (*n* = 247). All patients were evaluated monthly for the need for macular laser according to protocol-specified criteria including central foveal thickness ≥ 250 *μ*m. In RISE, 18% of sham patients gained ≥ 15 letters versus 45% of 0.3 mg (*P* < 0.0001; difference versus sham adjusted for randomisation stratification factors, 24%) and 39% of 0.5 mg IVR patients (*P* < 0.001; adjusted difference, 21%). In RIDE, 12% of sham patients gained ≥ 15 letters versus 34% of 0.3 mg patients (*P* < 0.0001; adjusted difference, 21%) and 46% of 0.5 mg IVR patients (*P* < 0.0001; adjusted difference, 33%). IVR patients underwent significantly fewer macular laser procedures and ocular safety was consistent with other IVR studies (endophthalmitis in 4 IVR patients). Guidance by NICE states that up to 0.5 mg of IVR should be given monthly and continued until maximum visual acuity is reached, defined as stable visual acuity for three consecutive months. The FDA has approved the lower dose of 0.3 mg, once monthly injections of IVR for DMO. The discrepancy in the approved dosages relates to cost effectiveness. It appears that the lower dose of 0.3 mg has a similar success profile to the higher 0.5 mg dose; however the NHS is able to obtain the drug at a discounted rate through a patient access scheme and therefore prescribe up to 0.5 mg for the same cost [23].

Bevacizumab (IVB, Avastin; Genentech Inc.) is a full-length humanised antibody that binds all forms of VEGF-A. It is not licensed for intraocular use but is a much less costly alternative with a good evidence base. The Decision Support Unit (DSU) is commissioned by NICE to provide research to support the Institute's Technology Appraisal Programme. As such, they have evaluated the efficacy of IVB for the treatment of DMO [23]. Based on seven RCTs, they conclude that efficacy measures for visual acuity (BCVA ETDRS ≥ 15 letters) favoured IVB compared with laser therapy, although the effect size is diminished as follow-up time is increased. BCVA LogMAR scores indicate that only longer-term treatment is advantageous over laser therapy, whilst changes in central retinal thickness did not indicate that IVB confers a sustained advantage over laser therapy.

Of note, some trials were not included within the report such as the 24-month data from the BOLT study [24]. This was a prospective, masked, single centre, two-arm trial with subjects randomised to either IVB or macular laser therapy. At two years, the IVB group gained a mean of 8.6 ETDRS letters, whereas the laser group lost a mean of 0.5 ETDRS letters (*P* = 0.005). Mean reduction in central retinal thickness was 146 *μ*m in the IVB arm versus 118 *μ*m in the laser arm. It is likely that the use of IVB is limited in NHS patients mainly as a result of NICE guidance in favour of an alternative therapy [23].

Aflibercept (IVA, Eyelea; Regeneron/Bayer HealthCare) is a recent addition to the anti-VEGF class. It is a fully human, soluble VEGF receptor fusion protein that targets all forms of VEGF-A and placental growth factor. The da Vinci Study Group published one-year outcomes comparing different doses and dosing regimens of IVA with macular laser in patients with DMO [25]. The study was an industry sponsored double-masked, multicentre phase 2 clinical trial which randomised patients into one of five groups. Mean improvements in BCVA in the IVA groups at week 52 were 11.0, 13.1, 9.7, and 12.0 letters for different dosing regimens (0.5 mg every 4 weeks, 2 mg every 4 weeks, 2 mg every 8 weeks after 3 initial monthly doses, and 2 mg as needed after 3 initial monthly doses, resp.) versus −1.3 letters for the laser group (*P* ≤ 0.0001 versus laser). Mean reductions in central retinal thickness in the IVA groups at week 52 were −165.4 *μ*m, −227.4 *μ*m, −187.8 *μ*m, and −180.3 *μ*m versus −58.4 *μ*m for laser (*P* < 0.0001 versus laser).

Two similarly designed, double-masked, randomised phase 3 trials (VISTA^DME^ and VIVID^DME^) compared IVA (2 mg every 4 weeks (2q4) and 2 mg every 8 weeks (2q8) groups) with laser treatment for DMO [26]. Mean BCVA gains from baseline to week 52 in the IVA 2q4 and 2q8 groups versus the laser group were 12.5 and 10.7 versus 0.2 letters (*P* < 0.0001) in VISTA and 10.5 and 10.7 versus 1.2 letters (*P* < 0.0001) in VIVID. Similarly, mean reductions in central retinal thickness were 185.9 and 183.1 versus 73.3 *μ*m (*P* < 0.0001) in VISTA and 195.0 and 192.4 versus 66.2 *μ*m (*P* < 0.0001) in VIVID. Incidences of ocular and nonocular adverse events were similar across treatment groups.

Further studies comparing IVA, IVR, and IVB are being performed by the DRCR.net group with expected reporting date January 2016 [27]. IVA has very recently been approved by the European Commission for the treatment of visual impairment as a result of DMO. It is due for NICE technology appraisal in June 2015. The recommended dose of IVA for the treatment of DMO is 2 mg. Treatment is initiated with one injection per month for five consecutive doses, followed by one injection every two months without any requirement for monitoring between injections. After the first 12 months of treatment, the treatment interval may be extended based on visual and anatomic outcomes.

A full discussion with regard to the role of intravitreal steroids or laser photocoagulation is beyond the scope of this report. It is worth mentioning that a NICE technology appraisal recommends fluocinolone acetonide intravitreal implant (Iluvien, Alimera Sciences Inc.) for pseudophakic patients with DMO unresponsive to other treatment options [28]. Ozurdex (dexamethasone intravitreal implant) has recently received a European marketing license for the use in “adult patients with visual impairment due to DMO who are pseudophakic or who are considered insufficiently responsive to or unsuitable for noncorticosteroid therapy.” NICE technology appraisal is due in April 2015. Steroid implants may reduce the frequency of intravitreal injections but have recognised complications of increased intraocular pressure and cataract formation.

Overall, there is high quality evidence that anti-VEGF drugs provide benefit compared with other therapeutic options for DMO. This is supported by Systematic Reviews published in the Cochrane Library and a similar review produced by the American Academy of Ophthalmology [29]. This concluded that “anti-VEGF pharmacotherapy, delivered by intravitreal injection, is reasonably safe and effective in the treatment of DME.” A statement by the Royal College of Ophthalmologists in their published diabetic retinopathy guidelines confirms that “anti-VEGF injections are considered the new gold standard of therapy for eyes with centre-involving macular oedema and reduced vision” [30, 31]. Future research should now compare these drugs and treatment regimens to help refine clinical care pathways.

## 3. AMD

Recently, the targeted therapy of VEGF has revolutionised the treatment of neovascular AMD. In 2004, the U.S. Food and Drug Administration approved the first treatment targeting VEGF, pegaptanib sodium injection (Macugen; EyeTech, New York, NY). This is a pegylated aptamer which binds to the 165 isoform of VEGF.

The VISION (VEGF Inhibition Study in Ocular Neovascularisation) study was a prospective randomised double-masked trial to assess the benefit of treating early subfoveal CNV secondary to AMD with pegaptanib sodium [7]. It randomised 1186 subjects to sham versus 3 doses of the drug, with each receiving an injection every 6 weeks. All 3 pegaptanib groups showed efficacy over sham treatment. 70% of those treated with the lowest (0.3 mg) dose avoided 3 or more lines of visual loss at 1 year, compared to 55% of controls [7]. VA was maintained in the 2nd year of the study [32].

In 2006, the antibody fragment, ranibizumab, was approved for use in neovascular AMD by the FDA. The landmark ANCHOR and MARINA studies aimed to access the efficacy of ranibizumab in both classic and minimally classic/occult neovascular AMD, respectively [11]. ANCHOR was a 2-year, randomised, double-blind trial comparing ranibizumab with PDT in predominantly classic subfoveal CNV [11, 33]. Patients had to be over 50, BCVA 20/40 to 20/320, and with a lesion size of less than 5400 microns. 423 subjects were randomised into 3 groups (3 monthly PDT and monthly sham intravitreal injections; monthly 0.3 mg ranibizumab with 3 monthly sham PDT; monthly 0.5 mg ranibizumab with 3 monthly sham PDT). After 1 year, 94.3% of 0.3 mg ranibizumab and 96.4% of the 0.5 mg ranibizumab groups lost less than 15 letters compared to 64.3% of those in the PDT group (*P* < 0.001) [11]. VA improved by 15 letters or more in 35.7% of the 0.3 mg group and 40.3% of the 0.5% group, versus 5.6% of the PDT group (*P* < 0.001)  [11]. These improvements were maintained at 2 years [33]. On average, VA improved over 8.1 to 10.7 letters from baseline, compared to an average loss of 9.8 letters in the PDT group. The anatomical lesion characteristics also improved in the ranibizumab group versus the control group. It concluded that both doses of ranibizumab were superior to PDT for classic neovascular AMD. Its sister study, the MARINA trial, was a similar 2-year multicentre, randomised, double-blind, placebo-controlled study comparing ranibizumab versus sham in minimally classic or occult CNV [11]. Patients had to be over 50, BCVA 20/40 to 20/320, and with a lesion of less than 12 disc diameters. 716 subjects were randomised to monthly sham, 0.3 mg ranibizumab, and 0.5 mg ranibizumab injections. At 1 year, 94.5% of the 0.3 mg group and 94.6% of the 0.5 mg group lost less than 15 letters, compared to 62.2% of the placebo group. VA improved by 15 letters in 24.5% of the 0.3 mg group and 33.8% of the 0.5 mg group, versus 5% of the sham group. The average improvement in VA was 6.5 letters in the 0.3 mg group and 7.2 letters in the 0.5 mg group, compared to a loss of 10.4 letters in the sham group. These improvements were maintained at 2 years. Both studies demonstrated that ranibizumab was effective at treating both classic and occult neovascular AMD.

Following the encouraging results of ANCHOR and MARINA, focus then shifted to investigating potential dosing regimens for ranibizumab in an attempt to reduce the treatment burden of monthly injections. The PIER study was a 2-year RCT involving 184 patients given quarterly injections after an initial 24-month period with a subsequent phase of monthly injections in the latter part of year 2 [34]. It found that although average VA improved in the treatment groups for the first 3 months, there was a gradual decline in VA (approximately 2 letters) from months 4 to 24 when on quarterly dosing. This compared poorly to the VA stabilisation seen in the ANCHOR and MARINA trials. The EXCITE trial then directly compared monthly versus quarterly ranibizumab injections over 1 year [35]. It again found that VA improved in both regimes over the first 3 months and declined in the quarterly groups over the next 9 months. Average letters gained at 1 year were 8.3 letters in the monthly group, 4.9 letters in the 0.3 mg group, and 3.8 letters in the 0.5 mg group. This further supported the notion that although quarterly injections improved VA in neovascular AMD, it was not as effective as monthly injections. The PrONTO (prospective optical coherence tomography imaging of patients with neovascular AMD treated with intraocular ranibizumab) trial was a small, nonrandomised, uncontrolled, open-label study which used OCT to vary ranibizumab dosing following a 3-month loading phase [36]. Thirty-seven patients were retreated if there was persistence or increase of intraretinal fluid and decrease in VA of 5 or more letters or a new haemorrhage/area of CNV. Over 2 years, it found an average improvement of 11.1 letters, with a mean number of injections of 9.9. These results were comparable to ANCHOR and MARINA but were limited by the study design and small sample size. The SUSTAIN (safety and efficacy of a flexible dosing regimen of ranibizumab in neovascular age-related macular degeneration) study was a larger, 1 year single arm study which again involved PRN ranibizumab dosing following a 3-month loading phase [37]. Five hundred and thirteen patients were recruited, with parameters for retreatment being loss of more than 5 letters or increase of 100 microns in CRT. Mean BCVA was +5.8 letters at month 3, decreasing to +3.6 letters at 12 months. Average number of injections after loading was 2.7. This suggested that although VA improvement does decline slightly on PRN dosing, it may not worsen as much as the PIER study had reported. This paved the way for the HARBOR study [38]. This was a randomised, double-blind treatment-controlled study. 1098 patients were randomised to receive 0.5 mg or 2 mg ranibizumab on a monthly or PRN basis. Patients had to be over 50 with CNV < 12 disk diameters and BCVA 20/40 to 20/320. At 12 months, it found that the average gain in VA letters was +10.1 (0.5 mg monthly), +9.2 (2 mg monthly), +8.2 mg (0.5 mg PRN), and +8.6 mg (2 mg PRN) [38]. The mean change from baseline in CRT in the 4 groups was −172.0 *μ*m, −161.2 *μ*m, −163.3 *μ*m, and −172.4 *μ*m, respectively. The PRN groups required an average of 6.9–7.7 injections over the year. This study demonstrated that monthly doses of 0.5 mg ranibizumab produce the optimum visual results for patients with neovascular AMD, although PRN dosing does lead to clinically meaningful visual improvement not significantly different than monthly dosing. It also showed that quadrupling the dose does not improve VA results. Some clinicians support a “treat and extend” regime, which involves treating monthly until the macula is dry and then incrementally increasing time between injections whilst the macular remains dry [39, 40]. This seems to lead to stabilised VA with a reduction in injections but has not been assessed with a prospective, randomised controlled trial.

In 2004, intravenous bevacizumab was approved for the treatment of metastatic colon cancer, and this leads to a number of ophthalmologists using the drug off-label as an intravitreal injection. This offered a cheaper alternative to ranibizumab, although there were concerns over its safety and lack of trial data. In response to this, the CATT (Comparison of AMD Treatments Trials) and IVAN (Lucentis and Avastin effective in treating wet AMD) studies aimed to compare the safety and efficacy of using ranibizumab versus bevacizumab [41, 42]. The CATT trial was a multicentre, randomised trial involving 1208 subjects comparing the efficacy and side effects of monthly or PRN regimes of ranibizumab 0.5 mg and bevacizumab 1.25 mg [41]. At 1 year, CATT could not demonstrate that PRN bevacizumab was not inferior to monthly ranibizumab (RBZ monthly: +8.5 letters; BVZ monthly: +8 letters; RBZ PRN: +6.8 letters; BVZ PRN: +5.9 letters), although anatomically RBZ led to a greater decrease in CRT. Whilst it found that there was no difference in rates of death, stroke, or myocardial infarction, it did find that there was increased rate of hospitalization in the BVZ group (24.1% versus 19%). At 2 years, mean gain was again similar between the 2 groups, although monthly dosing performed better than PRN dosing. Rates of death and thrombotic events were the same, although numbers of patients with “one or more serious systemic adverse events” was higher in the BVZ group (39.9% versus 31.7%) [42]. This persisted even when previously recognised anti-VEGF adverse events were removed. IVAN was a smaller, similarly designed NHS-funded trial, recruiting 628 patients [42]. At 2 years, it found that BCVA was similar between the RBZ and BVZ groups and between the monthly and PRN regimes, although the primary outcome of BVZ being noninferior to RBZ was not met [42]. Pooled safety estimates of both trials found that there was no difference in death or thrombotic events between the 2 drugs but risk of systemic adverse events was higher in the BVZ group. Unexpectedly, it also found that the risk of death was higher in the PRN regime versus monthly regime. The authors suggested that this may be due to an immunological reaction and that further investigation was needed.

In 2011, aflibercept (VEGF Trap-Eye) was approved for use by the FDA. This is a fusion protein that binds to all isoforms of VEGF, with greater affinity than RBZ and BVZ. There was a hope that this increased VEGF-binding activity would decrease the frequency of injections. This prompted the VIEW 1 and 2 studies. These were 2 paired, multicentre, double-blind RCTs [43]. 2419 patients were randomised to aflibercept 0.5 mg monthly; aflibercept 2 mg monthly; aflibercept 2 mg two monthly (after loading phase); and RBZ 0.5 mg monthly. The study found that at year 1, all aflibercept groups were noninferior to the RBZ monthly groups, with the average BCVA of all 3 within 0.5 letters of the RBZ group [44]. Between year 1 and year 2, all the groups were changed from a fixed regimen to a variable regimen requiring at least quarterly dosing (capped PRN) [retreatment if loss of 5 letters, new or persistent fluid on OCT, increase in CRT of 100 microns, or new haemorrhage/CNV]. They found no difference between the groups, although there was a small decrease in BCVA letters gained during the 2nd year, in keeping with previous studies [43]. The groups needed 4.1–4.7 injections in the 2nd year. There were no differences in serious adverse events between any of the groups. The study showed that two monthly aflibercept gave equivalent VA results to monthly RBZ over 2 years, whilst needing 5 fewer injections. It did not however compare the groups to fixed-dosing RBZ or aflibercept over the full 2 years, which may have given better BCVA results.

With regard to future treatments, there are numerous new drugs for AMD going through phase II studies [45]. Among them is Fovista, a compound which inhibits platelet-derived growth factor from binding to pericytes. This would potentially increase the effectivity of anti-VEGF drugs. A recent phase 2 study showed that 6 monthly Fovista injections in combination with monthly RBZ were 60% more effective than RBZ alone [46]. This is currently undergoing phase III trials. Also of note is potential topical anti-VEGF agents, which would eliminate many of the risks or burden of regular intravitreal injections. PanOptica are currently testing PAN-90806, a topical small molecule VEGF antagonist in wet AMD.

SAILOR (Safety Assessment of Intravitreous Lucentis fOR AMD) investigated RBZ in a large population of 4300. Results were determined by angiography surveying subfoveal CNV secondary to AMD. This study showed RBZ was safe and well tolerated in a large population and a low risk of thromboembolism.

HORIZON (An Open-Label Extension Trial of Ranibizumab for Choroidal Neovascularization Secondary to Age-Related Macular Degeneration) was a 2 year study investigating the long term safety and efficacy in 853 patients completing the controlled treatment phase of 1 of the 3 2-yr clinical trials (ANCHOR, MARINA & FOCUS). Incidence of serious ocular and non-ocular adverse events over the 2 yrs were low and consistent with previous phase 3 studies.

SANA (Systemic Avastin for Neovascular AMD) examined the short term safety of systemic BVZ in 9 patients with subfoveal CNV and BCVA letter scores of 70 to 20 and CRT > 300 microns. Safety was measured by, change in VA scores, OCT measurement, fluorescein angiography, and indocyanine green angiography. The study proposed that BVZ was effective and safe for all patients over 24 months whilst acknowledging the study size.

ABC (Avastin (BVZ) for choroidal neovascular age-related macular degeneration) was a double masked randomised controlled trial with two parallel treatment groups IVT BVZ versus PDT (for classic) or pagaptanib (for occult). 131 patients were researched with 126 remaining for analysis. The proportion of patients gaining/losing > 15 letters at 1 yr demonstrated BVZ being more effective than standard care with low rates of adverse events.

## 4. RVO

Retinal vein occlusion (RVO) is a common cause of visual loss in the United Kingdom and is the second most common retinal vascular disease after diabetic retinopathy [47, 48]. RVOs can be divided into two categories depending on the site of obstruction: central RVO (CRVO) when occlusion involved the whole central retinal vein and branch RVO (BRVO) when the occlusion involves only one branch of the central retinal vein [49].

BRVO occurs 2-3 times more commonly than CRVO [50, 51]. In the Beaver Dam Eye Study the 15-year cumulative incidence of CRVO and BRVO was 0.5 and 1.8%, respectively [52].

The pathogenesis of RVO is multifactorial with thrombus formation being the primary cause but other possible causes are external compression or disease of the vein wall, for example, vasculitis. BRVO occurs at arteriovenous crossing sites [53]. CRVO is caused by external compression of the central retinal vein, which shares a common fibrous sleeve with the vein [54]. BRVO and CRVO typically occur in middle aged and elderly patients (i.e., over age of 50 years) with equal sex distribution. CRVO is classically characterised by disc oedema, increased dilatation and tortuosity of all retinal veins, widespread of deep and superficial haemorrhages, cotton wool spots, retinal oedema, and capillary nonperfusion. In less severe forms the disc oedema may be absent. BRVOs have similar features except that they are confined to a portion of the fundus.

The main cause of visual loss in RVO is macular oedema.

Visual outcome of CRVO depends on the visual acuity at presentation. Eyes with initial visual acuity of 6/12 (20/40) or better have a better prognosis for retaining good vision than those with worse vision. In BRVOs 50% of untreated eyes retain vision of 6/12 or better whilst 25% will have vision < 6/60 [50]. Up to 34% of nonischaemic CRVO convert to ischaemic forms within 3 years.

VEGF is produced by retinal pigmented epithelial cells, endothelial cells, Muller cells, and other ocular tissues in response to retinal hypoxia and binds to specific receptors on endothelial cells acting as a proangiogenetic factor. This in turn leads to neovascularization and vascular hyperpermeability with subsequent breakdown of the blood-retina barrier and macular oedema [55].

Until recently the standard of care for macular oedema secondary to BRVO was macular grid laser [47, 48]. Macular laser photocoagulation is not recommended for the treatment of macular oedema in CRVO because it does not guarantee a significant improvement in visual acuity. Panretinal photocoagulation of the ischemic retina is indicated both in BRVO and in CRVO if iris, retinal, or disc neovascularization is present.

Currently available anti-VEGF agents (ranibizumab, aflibercept, and bevacizumab) have been applied successfully in reducing macular oedema due to RVO.

In May 2013 NICE recommended ranibizumab (Lucentis, Novartis) as a possible treatment for some people who have sight problems because of macular oedema caused by retinal vein occlusion. Indications include macular oedema in CRVO or in BRVO only if previous grid laser has failed or laser is not suitable due to the extent of haemorrhage.

Two randomized, double-masked, multicenter phase 3 trials, the CRUISE (central retinal vein occlusion) and BRAVO (branch retinal vein occlusion) studies, evaluated the efficacy of ranibizumab, compared with a sham procedure, for treating visual impairment caused by macular oedema secondary to BRVO and to CRVO, respectively [56, 57].

The BRAVO (*n* = 397) and CRUISE (*n* = 392) trials were both 3-armed RCTs carried out at multiple centres in the USA. Patients were eligible if they had foveal-involved macular oedema from a CRVO or BRVO occurring within 12 months of study entry, BCVA of 20/40 to 20/320 (in CRVO) and to 20/400 (in BRVO), and centre subfield thickness (CST) ≥ 250 *μ*m.

Patients were randomised equally to sham injection, monthly intraocular ranibizumab 0.3 mg, or monthly intraocular ranibizumab 0.5 mg. Patients entered a 6-month treatment phase during which monthly injections were given.

In the treatment phase of BRAVO, patients in both the sham injection and ranibizumab groups could receive grid laser photocoagulation for rescue treatment from 3 months.

In both BRAVO and CRUISE, the treatment phase was followed by a 6-month observation phase during which all groups could receive ranibizumab as needed.

Patients in the observation phase of BRAVO (but not CRUISE) could receive grid laser photocoagulation for rescue treatment for 3 months (i.e., at month 9 of the study). The final treatment in both BRAVO and CRUISE was given at month 11, with a final study visit at month 12. Patients who completed the 12-month BRAVO and CRUISE trials could enter an open-label extension study (HORIZON).

For patients enrolled in the CRUISE study baseline characteristics were well balanced among the three groups; the mean age was 68 years, mean BCVA was 20/100, the mean time from diagnosis of CRVO was 3.3 months, and the mean center point thickness (CPT) was 685 *μ*m. At 6 months, the primary endpoint, mean change from baseline BCVA letter score, was 12.7 and 14.9 in the 0.3 mg and 0.5 mg ranibizumab groups and 0.8 in the sham group (*P* < 0.0001). The percentage of patients who gained ≥ 15 letters in BCVA was 46.2% (0.3 mg) and 47.7% (0.5 mg) in the ranibizumab groups and 16.9% in the sham group (*P* < 0.0001). The percentage of patients with a Snellen equivalent BCVA of 20/40 or better was 43.9% (0.3 mg) and 46.9% (0.5 mg) compared with 20.8% in the sham group (*P* < 0.0001). The percentage of patients with a Snellen equivalent BCVA of 20/200 or worse was 15.2% (0.3 mg) and 11.5% (0.5 mg) compared with 27.7% in the sham group (*P* < 0.005). Based upon the NEI VFQ-25 survey, patients who received ranibizumab felt they had greater improvement (improvement from baseline in NEI VFQ score: 7.1, 0.3 mg; 6.2, 0.5 mg; 2.8, sham). There was greater reduction of macular edema in the ranibizumab groups because CPT was reduced by 433.7 *μ*m (0.3 mg) and 452.3 *μ*m (0.5 mg) compared to 167.7 *μ*m in the sham group. The percentage of patients with CPT ≤ 250 *μ*m at 6 months was 75.0% (0.3 mg), 76.9% (0.5 mg), and 23.1% (sham, *P* < 0.0001). This study demonstrated that six sessions of monthly injections of 0.3 mg or 0.5 mg reduced macular oedema and provided substantial visual benefit in patients with CRVO.

Baseline characteristics for those involved in the BRAVO study were well balanced among the three groups; mean BCVA was 20/80, the mean time from diagnosis of BRVO was 3.5 months, and the mean CPT was 520 *μ*m. Starting at month 3, patients were eligible for grid laser treatment if hemorrhages had cleared sufficiently to allow safe application of laser and the following criteria were met: Snellen equivalent BCVA ≤ 20/40 or mean CST ≥ 250 *μ*m, and compared with the visit 3 months before the current visit, the patient had a gain of <5 letters in BCVA or a decrease of <50 *μ*m in mean CST. If rescue laser was not given at month 3, the same criteria were applied at month 4, and if rescue laser was not given at month 4, the criteria were applied at month 5.

At month 6, the primary endpoint, mean change from baseline BCVA letter score, was 16.6 and 18.3 in the 0.3 mg and 0.5 mg ranibizumab groups and 7.3 in the sham group (*P* < 0.0001). The percentage of patients who gained ≥ 15 letters in BCVA was 55.2% (0.3 mg) and 61.1% (0.5 mg) in the ranibizumab groups and 28.8% in the sham group (*P* < 0.0001). The percentage of patients with a Snellen equivalent BCVA of 20/40 or better was 67.9% (0.3 mg) and 64.9% (0.5 mg) compared with 41.7% in the sham group (*P* < 0.0001). The percentage of patients with a Snellen equivalent BCVA of 20/200 or worse was 1.5% (0.3 mg) and 0.8% (0.5 mg) compared with 9.1% in the sham group (*P* < 0.01). Based upon the NEI VFQ-25 survey, patients who received ranibizumab felt they had greater improvement (improvement from baseline in NEI VFQ score: 9.3, 0.3 mg; 10.4, 0.5 mg; 5.4, sham). There was greater reduction of macular oedema in the ranibizumab groups because CPT was reduced by 337.3 *μ*m (0.3 mg) and 345.2 *μ*m (0.5 mg) compared to 157.7 *μ*m in the sham group. The percentage of patients with CPT ≤ 250 *μ*m at month 6 was 91% (0.3 mg), 84.7% (0.5 mg), and 45.5% (sham, *P* < 0.0001). More patients in the sham group (54.5%) received rescue grid laser therapy than in the 0.3 mg (18.7%) or 0.5 mg (19.8%) ranibizumab groups. There were no safety signals identified in either trial.

After the primary endpoint in the CRUISE and BRAVO trials, patients were evaluated every month and if study eye Snellen equivalent BCVA was ≤20/40 or mean CST was ≥250 *μ*m, they received an injection of ranibizumab; patients in the ranibizumab groups received their assigned dose and patients in the sham group received 0.5 mg. In patients with CRVO, the mean number of ranibizumab injections during the observation period was 3.9, 3.6, and 4.2 in the 0.3 mg, 0.5 mg, and sham/0.5 mg groups; and the percentage of patients that did not receive any injections during the observation period was 7.0, 6.7, and 4.3, respectively [56]. At month 12 in the ranibizumab groups, the improvement from baseline in ETDRS letter score was 13.9, very similar to the month 6 results, indicating that vision is well maintained when injections are given only if there is recurrent or residual macular oedema. Patients in the sham group showed substantial improvement during the observation period when they were able to receive ranibizumab; improvement from baseline in letter score was 0.8 at month 6 and 7.3 at month 12. The percentage of patients who had an improvement from baseline BCVA letter score ≥ 15 at month 12 was 47.0% (0.3 mg) and 50.8% (0.5 mg) in the ranibizumab groups, almost identical to the month 6 results. In the sham group, 33.1% of patients improved from baseline ≥ 15 in letter score at month 12 compared to 16.9% at month 6. At month 12, 43% of patients in the two ranibizumab groups had a Snellen equivalent BCVA of 20/40 compared to 35% in the sham/0.5 mg group.

In patients with BRVO, the mean number of ranibizumab injections during the observation period was 2.9, 2.8, and 3.8 in the 0.3 mg, 0.5 mg, and sham/0.5 mg groups; and the percentage of patients that did not receive any injections during the observation period was 17.2, 20.0, and 6.5, respectively [57]. At month 12 in the ranibizumab groups, the improvement from baseline in ETDRS letter score was 16.4 (0.3 mg) and 18.3 (0.5 mg), very similar to the month 6 results, indicating that vision is well maintained when injections are given only if there is recurrent or residual macular oedema. Patients in the sham group showed substantial improvement during the observation period when they were able to receive ranibizumab; improvement from baseline in letter score was 7.3 at month 6 and 12.1 at month 12. The percentage of patients who had an improvement from baseline BCVA letter score ≥ 15 at month 12 was 55.2% (0.3 mg) and 61.1% (0.5 mg) in the ranibizumab groups, almost identical to the month 6 results. In the sham group, 43.9% of patients improved from baseline ≥ 15 in letter score at month 12 compared to 28.8% at month 6. At month 12, 67.9% (0.3 mg) and 64.4% (0.5 mg) of patients in the ranibizumab groups had a Snellen equivalent BCVA of 20/40 compared to 56.8% in the sham/0.5 mg group. Thus, in both CRUISE and BRAVO, patients in the sham groups showed a substantial improvement in vision during the second 6 months when they were able to receive ranibizumab as needed, but their vision at month 12 was not as good as that in patients in the ranibizumab groups.

For patients who entered the open-label extension study (HORIZON), ranibizumab 0.5 mg was given at intervals of at least 30 days. 67% of patients from BRAVO and 60% of patients from CRUISE completed month 12 of HORIZON. The primary outcome for the HORIZON extension study was mean change from HORIZON baseline in BCVA score up to 24 months. The manufacturer presented results from the first 12 months. From the BRAVO trial baseline, patients receiving sham (plus ranibizumab) and those receiving 0.5 mg ranibizumab had mean gains in BCVA score of 15.6 letters and 17.5 letters, respectively. From the CRUISE trial baseline, patients receiving sham (plus ranibizumab) and those receiving 0.5 mg ranibizumab had mean gains in BCVA score of 7.6 and 12.0 letters, respectively (no confidence intervals reported).

Adverse events were reported at 6 months and 12 months in both BRAVO and CRUISE trials and for a further 12 months' follow-up in the HORIZON extension study. In BRAVO, at 6 months there were 7 ocular adverse events (5.4%) in the ranibizumab group compared with 17 (13%) in the sham group, excluding occurrences of raised intraocular pressure. Nonocular serious adverse events (potentially related to vascular endothelial growth factor [VEGF] inhibition) at 6 months were higher in the ranibizumab group (5 events [3.8%]) than in the sham group (1 event [0.8%]). In CRUISE, at 6 months there were 13 ocular adverse events (10.1%) in the ranibizumab group compared with 25 (19.4%) in the sham group, excluding occurrences of raised intraocular pressure. In CRUISE, nonocular serious adverse events (potentially related to VEGF inhibition) were similar in both the ranibizumab and sham groups (3 [2.3%] and 2 [1.6%], resp.). The most common adverse event reported in BRAVO and CRUISE at 12 months was cataract, with 8 (6.2%) and 9 (7%) instances associated with ranibizumab treatment, respectively; in the sham (plus ranibizumab) group, 3 (2.6%) and 2 (1.8%) instances of cataract were reported for the treatment period of 6 to 12 months. Instances of raised intraocular pressure were reported in both BRAVO and CRUISE at 6 months but were academic in confidence and therefore not reported here. In the HORIZON extension study, the incidence of any adverse event in the sham (plus ranibizumab) and ranibizumab groups was 2.2% and 5.8%, respectively, for the patients (with BRVO) recruited from BRAVO and 5.2% and 3%, respectively, for the patients (with CRVO) recruited from CRUISE.

In February 2014 NICE recommended aflibercept (Eylea, Bayer) as a possible treatment for people with sight problems caused by macular oedema from central retinal vein occlusion.

Aflibercept for CRVO was studied in particular in the COPERNICUS and GALILEO trials, both phase III clinical trials for CRVO-related macular oedema [58].

COPERNICUS (vascular endothelial growth factor trap-eye for macular edema secondary to central retinal vein occlusion) was a randomised, double-blind, multicentre trial conducted in 6 non-European countries.

GALILEO was a randomised, double-blind, multicentre trial conducted in 10 European and Asian-Pacific countries.

In both trials the included patients had been diagnosed less than 9 months before the start of the trial and they had not received previous treatment for CRVO. The trials had identical designs and criteria for the first 6 months: both were randomized, multicentre, and double-masked, and both included patients with central retinal thickness (CRT) of ≥250 *μ*m and BCVA of 20/40 and 20/320.

From week 0 to week 24, patients in the intervention group received intravitreal aflibercept injection (IAI) every 4 weeks and patients in the comparator group received a sham injection every 4 weeks.

The primary end point was the proportion of those patients who gained three or more lines, and the key secondary end points were total change in BCVA and CRT.

The primary end points were assessed at 6 months and demonstrated that 23.3% of the sham-treated patients and 49.1% of the IAI patients in COPERNICUS, with 15 and 62% of respective patients in GALILEO, experienced a gain of three lines or more.

Mean change in VA for COPERNICUS at 6 months was four letters for the sham-treated group and +17.3 letters for the IAI-treated group. Mean change in VA for GALILEO at 6 months was +3.3 letters for the sham-treated group and +18.0 letters for the IAI-treated group. In addition, at 6 months, the proportion of patients for COPERNICUS without retinal edema was 15.3% in the sham-treated eyes and 74.5% in the IAI-treated eyes. These studies also demonstrated a rapid response in visual and anatomical outcome that was more significant if treated within 2 months of the diagnosis. Early treatment with IAI results in better visual and anatomical results than delaying treatment, and these results were regardless of perfusion status of the retina at the time of treatment.

The COPERNICUS trial was extended to 100 weeks and allowed all patients to then be eligible for IAI 2q4 pro re nata (PRN) treatment protocol [59].

The GALILEO trial was to be extended to 76 weeks had the IAI group become the IAI 2q4 PRN group, but the sham-treatment group continued to be treated with sham until 52 weeks. After 52 weeks, the sham group was able to be treated with IAI 2q4 PRN. The retreatment criteria were an increase in CRT of >50 *μ*m from the lowest previous measurement; new or persistent cystic retinal changes or subretinal fluid or diffuse edema ≥ 250 *μ*m in the central subfield; a loss of ≥five letters from previous BCVA if associated with any increase in CRT; or a loss of ≥five letters between the current and most recent visit. Between 6 months and 1 year, for the COPERNICUS trial, the injection group received additional 2.7 injections while the sham/crossover group received 3.9 injections. For GALILEO, the final 6 months received an additional 2.5 injections.

COPERNICUS results at 100 weeks demonstrated sustained improvements in all parameters for the treatment group when converting to PRN treatment at 6 months and an additional improvement for sham treatment that was converted to PRN treatment at 6 months. Aflibercept was effective at reducing edema and improving vision, even when a delay in treatment takes place but outlines the likely irreversible visual damage that limits the visual recovery when treatment is delayed.

## 5. Myopic Choroidal Neovascularisation

Myopia is a common and multifactorial condition that affects 20–40% of the global population [60]. High myopia is classified as greater than −6 dioptres refractive error and commonly associated with an axial length of more than 26 mm [61–63]. It has a global prevalence of 0.5–5% [62]. Systemic associations of high myopia include prematurity and syndromes such as Down, Ehlers-Danlos, Knobloch, Marfan, Noonan, Pierre-Robin, and Stickler syndrome [61, 64].

Pathological myopia, also called degenerative myopia, is characterised by excessive elongation of the globe and associated pathological changes at the posterior pole such as tessellated fundus, posterior staphyloma, and myopic conus [60–62, 64]. It has a prevalence of 0.9–3.1% and studies have identified it as the primary cause for blindness in 7% and 12–27% of the population in Europe and Asia, respectively [60, 65].

Choroidal neovascularisation (CNV) is one of the complications of pathological myopia and occurs in 5.2–11.3% of patients with high myopia [60–63]. Clinically, myopic CNV may be associated with underlying myopic abnormalities such as focal chorioretinal atrophy or ruptures in the RPE-Bruch's membrane-choriocapillaris complex, also called “lacquer cracks” [61, 64]. Patients typically present with deteriorating visual acuity, central scotoma, or metamorphopsia [66].

Myopic CNV is characterised by the formation of abnormal blood vessels in the retina or under the retinal pigment epithelium (RPE), potentially penetrating Bruch's membrane into the subretinal space [60]. On slit-lamp biomicroscopy, a flat greyish membrane is observed, occasionally with a hyperpigmented border if chronic or recurrent [66]. As the CNV regresses, it leaves a fibrous pigmented scar called Fuchs' or Forster-Fuchs' spot as well as surrounding chorioretinal atrophy in later stages [66].

Fundus fluorescein angiography (FFA) is used in diagnosis in the acute stage, showing a “classic” CNV pattern. Optical coherence tomography (OCT) is used in monitoring. Myopic CNV lights up as a highly reflective area above the RPE, occasionally with minimal subretinal fluid [66].

The pathogenesis of myopic CNV is not yet fully understood but is thought to involve an imbalance of proangiogenic and antiangiogenic factors brought on by the mechanical stresses of a progressively elongating retina [66]. Genetic studies have also shown possible association of single nucleotide polymorphisms in the complement factor I (CFI) gene on chromosome 4 that encodes a protein involved in the alternative complement pathway [62, 66]. Alternatively, myopic CNV may occur de novo in patients with no previous signs of pathological myopia. Of note, in patients with preexisting myopic CNV, one study showed that 35% of fellow eyes go on to develop CNV within 8 years [63, 66].

Visual prognosis in myopic CNV is varied. Poor prognostic indicators include lower baseline visual acuity, age above 40 years, extensive chorioretinal atrophy, subfoveal location of the CNV, and lesion size above 400 *μ*m [60, 61, 63, 66].

Prior to the advent of antivascular endothelial growth factor (anti-VEGF) therapy, management of myopic CNV was largely based on laser photocoagulation for extrafoveal CNV and verteporfin photodynamic therapy for subfoveal CNV [61, 63, 66]. Neither has been able to show any consistent long-term visual benefit but anti-VEGF agents have recently been introduced with promising results [66].

Ranibizumab (Lucentis) is the only anti-VEGF therapy licensed for the treatment of myopic CNV and has shown great potential for visual gain and preventing irreversible retinal damage in phases II and III clinical trials REPAIR and RADIANCE (a randomized controlled study of ranibizumab in patients with choroidal neovascularization secondary to pathologic myopia), respectively [4, 66]. Bevacizumab (Avastin) has not been approved for intraocular use and therefore lacks both an established safety and efficacy profile. MYRROR, a phase III clinical trial, is currently ongoing to establish the role of aflibercept (Eylea) in the treatment of myopic CNV [66].

Based on the REPAIR and RADIANCE trials, Wong et al. have presented an anti-VEGF treatment algorithm for myopic CNV [66]. This algorithm involves a single initial intravitreal injection of an anti-VEGF agent, followed by subsequent “as needed” injections [66]. Follow-up is advised as monthly for two months and then 3-monthly in the first year [66]. At follow-up, symptoms and signs that warrant another anti-VEGF injection include reduced visual acuity, visual symptoms (e.g., metamorphopsia), or signs of disease activity on FFA or OCT [66]. Following the first year of monitoring, follow-up will be decided upon after discussion between the treating ophthalmologist and patient [66]. Further research and monitoring are yet to establish long-term outcomes and best management strategy.

## 6. Other Anti-VEGF Indications

Since their introduction into clinical practice, the list of possible uses for anti-VEGF therapy in ophthalmology has steadily expanded. Anti-VEGF agents have been utilized on an off-license basis for a vast array of other ocular pathologies ranging from external eye disease to ocular oncology and glaucoma. Indeed, a literature search in 2008 revealed 51 different ophthalmic diseases that had been treated with bevacizumab [67].

### 6.1. Anti-VEGF for Non-ARMD Associated CNV

The development of choroidal neovascular membranes may complicate any disease process which results in a defect in Bruch's membrane and can pose a threat to vision through leakage of fluid, haemorrhage, and subretinal fibrosis. Anti-VEGF agents are now widely used in the treatment of CNVM regardless of aetiology. In November 2013, NICE approved ranibizumab for the treatment of choroidal neovascularisation associated with pathological myopia in the UK. This decision was based upon evidence largely extracted from the RADIANCE (randomized controlled study of ranibizumab in patients with choroidal neovascularisation secondary to pathologic myopia) trial which compared ranibizumab with verteporfin photodynamic therapy (vPDT). This study showed superiority of ranibizumab over vPDT in terms of improvements in BCVA at month three, with improvements being sustained up to month twelve through further injections as required based on visual acuity or disease activity criteria [65, 68]. Other studies have shown a similar efficacy of ranibizumab and bevacizumab in the treatment of myopic CNV [69, 70]. Intravitreal anti-VEGF agents have also been successfully utilized in the treatment of CNV related to angioid streaks, uveitis, and trauma [71–73].

### 6.2. Anti-VEGF for Vascular Proliferative Retinal Diseases

Intravitreal injection of an anti-VEGF agent is now commonly performed as a pretreatment prior to pars plana vitrectomy for severe proliferative diabetic retinopathy. Several studies have demonstrated that a single dose of bevacizumab delivered in the weeks before surgery facilitates the dissection of fibrovascular membranes and reduces the likelihood of intraoperative and postoperative bleeding [74, 75]. Development or progression of tractional retinal detachment has however been reported following intravitreal injection of bevacizumab as an adjunct to pars plana vitrectomy which may be attributable to the contraction of fibrovascular membranes induced by the drug [76]. Regression and a decrease in vascular permeability of new vessels in uncomplicated proliferative diabetic retinopathy have also been demonstrated following the intravitreal injection of bevacizumab [77]. Reduction in the drive for VEGF production through pan-retinal laser photocoagulation however remains the first line treatment in the UK although anti-VEGF may have a role as an adjunctive therapy, particularly in cases of persistent vitreous haemorrhage [78, 79].

Retinopathy of prematurity (ROP) in its most severe form results in tractional retinal detachment secondary to fibrovascular proliferation and remains a leading cause of childhood blindness across the world. There has been much interest recently in the use of anti-VEGF agents to reduce the angiogenic drive that underlies the pathology of ROP. In their prospective randomized trial comparing intravitreal bevacizumab with conventional laser photocoagulation therapy, Mintz-Hittner et al. reported on behalf of the BEAT-ROP Cooperative Group a significant benefit of anti-VEGF in the treatment of stage 3+ zone 1 disease [80]. They also found that the development of peripheral retinal vessels continued after bevacizumab but not after laser therapy which may have a beneficial effect on conservation of visual field. Although relatively large, the BEAT-ROP study was not adequately powered to determine the safety of anti-VEGF in the developing child and some concerns remain regarding the potentially unknown long-term local and systemic side effects of these medications.

### 6.3. Anti-VEGF for Central Serous Retinopathy

Central serous retinopathy (CSR) is an idiopathic condition characterized by the accumulation of subretinal fluid at the macula. Although the majority of cases resolve spontaneously within six months, in some the fluid persists which can result in ongoing reduction in visual acuity and metamorphopsia. Intravitreal bevacizumab has been advocated by some as a possible treatment option in such cases of persistent CSR. Several small case series have reported significant reductions in central macular thickness and improvements in visual acuity in CSR patients treated with intravitreal bevacizumab [81, 82].

### 6.4. Anti-VEGF for Ocular Tumours

Anti-VEGF therapy is also being utilized in ocular oncology. Regression of choroidal metastases has been reported following treatment with both systemic and intravitreal bevacizumab [83–85]. Intravitreal bevacizumab has also been used by Mason III et al. in the treatment of radiation-induced macular oedema which may arise as a complication of plaque radiotherapy for choroidal melanoma [86]. Although a reduction in central retinal thickness was demonstrated amongst these patients, the effect was short-lived and associated with only marginal improvements in visual acuity. Intravitreal bevacizumab does not seem to halt the progression of choroidal melanoma in patients who have inadvertently received this drug on account of an initial misdiagnosis of CNVM [87].
